# Case Report: Effective use of eculizumab in treating recurrent atypical HUS following renal transplantation triggered by SARS-CoV-2 infection

**DOI:** 10.3389/fmed.2025.1515988

**Published:** 2025-11-05

**Authors:** Zhe Yang, Dongdong Chen, Xiaoyu Xu, Xiaoqing Yang, Wenzhi Du, Minrui Zhang, Xianduo Li, Jianning Wang

**Affiliations:** ^1^Department of Urology, The First Affiliated Hospital of Shandong First Medical University & Shandong Provincial Qianfoshan Hospital, Jinan, China; ^2^Shandong University, Jinan, China; ^3^Department of Pathology, The First Affiliated Hospital of Shandong First Medical University & Shandong Provincial Qianfoshan Hospital, Jinan, China; ^4^Shandong First Medical University, Jinan, China

**Keywords:** COVID-19, renal transplant, atypical hemolytic uremic syndrome, eculizumab, therapeutic

## Abstract

Atypical hemolytic uremic syndrome (aHUS) is a severe post-transplant complication associated with a high risk of graft loss and mortality. Based on its pathophysiological mechanism, aHUS can be classified into recurrent aHUS (reactivation post-transplantation) and *de novo* aHUS (new-onset disease after transplantation). This study reports a case of a 52-year-old kidney transplant recipient who developed recurrent aHUS 10 months post-transplantation, triggered by SARS-CoV-2 infection. Genetic analysis revealed mutations in complement-related genes CFHR and C3. Following the diagnosis, the patient received a course of eculizumab induction therapy (900 mg/week for the first 4 weeks), followed by long-term maintenance therapy (900 mg every 2 weeks). Significant clinical improvement was observed, including stabilization of blood pressure, normalization of hemoglobin and lactate dehydrogenase (LDH) levels, an increase in platelet count, and a urine output exceeding 2,000 mL per day. The patient continued regular follow-up after discharge, with stable graft function. This case highlights the potential role of SARS-CoV-2 and other infections as triggers for aHUS recurrence in kidney transplant recipients and further supports the efficacy of eculizumab in treating recurrent aHUS. Furthermore, this study underscores the importance of precise immune risk stratification and early intervention in the effective management of aHUS recurrence.

## 1 Introduction

Coronavirus disease 2019 (COVID-19) is a respiratory and systemic illness caused by infection with SARS-CoV-2. Although the disease predominantly impacts the lungs, it can extend to other organs, leading to complications such as acute kidney injury (1). The tissue damage associated with COVID-19 primarily stems from the host immune system's inflammatory response, characterized by hypercytokinemia and severe inflammation. This inflammatory cascade damages lung parenchymal cells, impairing oxygen exchange, and affects endothelial cells, causing endotheliitis, thrombotic events, and intravascular coagulation (2). Furthermore, COVID-19 can trigger thrombotic microangiopathy (TMA) through activation of the alternative and lectin complement pathways, potentially exacerbating disease progression (3).

TMA is characterized by thrombocytopenia and microangiopathic hemolytic anemia due to acute or chronic endothelial cell injury, often progressing to renal insufficiency (4). Atypical hemolytic uremic syndrome (aHUS) is a systemic, complement-mediated TMA and a severe complication following kidney transplantation, with an incidence of approximately 0.8%−14% (5). It carries a high risk of graft loss and even recipient mortality. Post-transplant aHUS in kidney transplant recipients is categorized into recurrent aHUS (post-transplant recurrence) and de novo aHUS (post-transplant onset). Recurrent aHUS typically manifests early in the postoperative period (6). While less common than *de novo* aHUS, recurrent aHUS is associated with a more severe impact on graft survival due to its propensity for recurrence, with up to one-third of transplanted kidneys potentially lost in affected patients (7). Currently, aHUS pathogenesis is believed to be primarily attributed to genetic variants in complement regulatory proteins or complement intrinsic proteins, or the presence of anti-complement factor H (CFH) antibodies. These predisposing factors, when combined with secondary triggers such as infections, pregnancy, or surgical procedures, create a “two-hit” mechanism that leads to aberrant activation of the complement alternative pathway and subsequent endothelial damage. Notably, over half of aHUS patients demonstrate detectable complement gene variants or CFH autoantibodies in clinical assessments (8).

Eculizumab is a recombinant, fully humanized monoclonal antibody that specifically targets human complement protein C5. By inhibiting the cleavage of C5 into C5a and C5b, eculizumab effectively prevents the formation of the membrane attack complex (MAC) and C5a anaphylatoxin, thereby mitigating endothelial injury (9). This complement inhibition has been established as an effective treatment for recurrent aHUS following renal transplantation. Compared to plasma exchange (PE), eculizumab offers superior disease control with an improved safety profile (10). This advancement holds promise for transforming aHUS from a critical threat to graft survival into a manageable condition through specialized treatment.

## 2 Report of case

**Figure 1:**
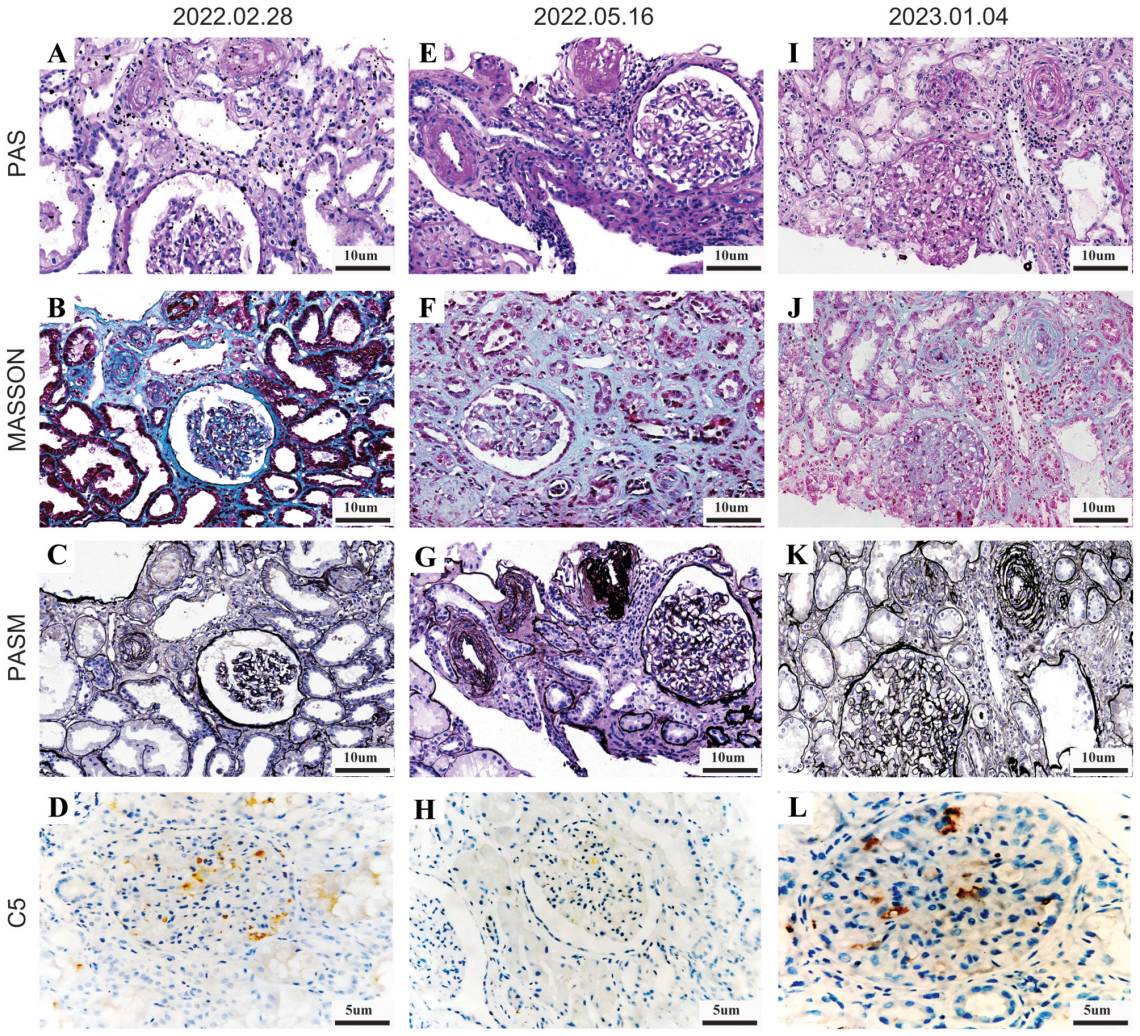
Biopsy specimen of the transplanted kidney from this patient. **(A–D)** Figures were obtained on February 28, 2022. The PAS-stained image **(A)** shows arterioles in the upper left corner with endothelial swelling and luminal constriction. Masson's trichrome stain **(B)** reveals endothelial swelling of arterioles, accompanied by focal plasma extravasation within the arteriolar walls. The PASM stain **(C)** demonstrates progressive luminal narrowing of arterioles leading to occlusion and wrinkling of the glomerular capillary loops. Immunohistochemical staining **(D)** indicates positive reactivity for C5 antibodies. **(E–H)** Figures were taken on May 16, 2022. The PAS **(E)**, Masson **(F)**, and PASM **(G)** stains show the absence of intramural endothelial edema or luminal narrowing in arterioles. However, interstitial fibrosis, arterial narrowing, and wrinkling of glomerular capillaries are noted. Immunohistochemical staining **(H)** shows negative expression of C5 antibodies. **(I–L)** Figures were obtained on January 4, 2023. The current biopsy images depict arterial endothelial edema across various stains, including PAS **(I)**, Masson **(J)**, and PASM **(K)** (top images), with observable arterial luminal narrowing and endothelial cell edema. Immunohistochemical staining **(L)** shows positive expression of C5 antibodies.

A 52-year-old male with kidney failure secondary to suspected aHUS underwent a kidney transplant. His medical history included hypertension and a mild increase in serum creatinine 4 years prior. He was diagnosed with TMA 3 years earlier due to oliguria, a rapid increase in serum creatinine, anemia, thrombocytopenia, and elevated lactate dehydrogenase (LDH) levels (>2,000 μmol/L). Following regular hemodialysis, his hemoglobin and platelet levels stabilized. The kidney was obtained from a donation after cardiac death. The patient's current flow panel-reactive antibody (PRA) class I/II levels were 6/0, with no donor-specific antibodies (DSA) and a negative flow cross-match. HLA matching with the donor was as follows: 0A, 1B, 1DR, 1DQ mismatch, 16 DRB1 3/4/5, and 13 DQA1/DQB1 eplet mismatch. He received induction therapy with intravenous anti-thymocyte globulin (2 mg/kg) and maintenance immunosuppression with tacrolimus (4 mg, bid), mycophenolate mofetil (720 mg, bid), and prednisone (16 mg, qd). Five days post-transplant, serum creatinine decreased to 200 μmol/L but did not fall further. This was accompanied by decreased hemoglobin and platelets and increased LDH levels. C5b-9 levels remained elevated at 346 ng/ml (normal range: 85.5 ± 21.1 ng/ml). A kidney biopsy revealed mild TMA (Figure 1, 2022.02.28). The patient underwent three sessions of single PE, resulting in improvement in laboratory parameters. Serum creatinine stabilized at 110 μmol/L, tacrolimus concentration was maintained at 6–8 ng/ml, and blood pressure was 130/80 mmHg. The patient was discharged in stable condition. During regular outpatient follow-ups over the next 3 months, all indicators remained stable. PRA review was negative, and C5b-9 levels decreased to 192 ng/ml. A scheduled renal biopsy showed no active lesions in the transplanted kidney (Figure 1, 2022.05.16).

At 10 months post-transplant, the patient presented with fever, cough, and sore throat. PCR testing for SARS-CoV-2 returned positive. At this time, the patient's serum creatinine had risen to 590 μmol/L, hemoglobin had dropped to 80 g/L, platelets had decreased to 21 × 10^Λ^9/L, and FK506 levels were 7.5 ng/ml. A CT scan indicated bilateral polysegmental viral pneumonia (Supplementary Figure 1). A biopsy of the transplanted kidney, performed on January 4, 2023, showed TMA-like changes (Figure 1). These findings, in conjunction with the patient's clinical history and serological results, were consistent with the diagnosis of recurrent aHUS. Sequencing analysis of disease-related genes revealed no suspicious variants linked to nephropathy; however, variants associated with aHUS, specifically in the C3 and CFHR5 genes, were identified (Supplementary Table 1).

Testing for HLA and non-HLA antibodies was negative, and the kidney biopsy results excluded rejection. ADAMTS13 activity was measured at 68.56% (typically <10% in thrombotic thrombocytopenic purpura). Additional serological tests, including ANCA, ANA, and antiphospholipid antibodies, were negative. The red blood cell fragmentation test showed that helmet-shaped red blood cells were greater than 3%, which supported the initial diagnosis of microangiopathic hemolytic anemia (MAHA). Complement C3 level was 0.780 g/L; Complement C4 level was 0.182 g/L; and C5b-9 level was 704 ng/mL (Supplementary material). In the absence of evidence for thrombotic thrombocytopenic purpura, antiphospholipid syndrome, or other systemic diseases such as systemic lupus erythematosus or ANCA-associated vasculitis, a diagnosis of aHUS was established (Table 1), likely triggered by SARS-CoV-2 infection. After antiviral therapy, the patient's SARS-CoV-2 test result turned negative, and lung symptoms improved. However, aHUS symptoms still recurred despite plasma exchange.

**Table 1:** Diagnostic criteria for TA-TMA.

**Biopsy with micro thrombosis evidence or meet the following five of seven laboratory or clinical indicators**
①The level of lactate dehydrogenase (LDH) was above the upper limit of normal value
②Proteinuria (random urinary protein exceeding the upper limit of normal values or random urinary protein/creatinine ratio ≥ 2 mg/mg)
③Hypertension (Age <18 years: blood pressure above the upper limit of normal reference values for healthy individuals of the same age, gender, and height; Age ≥ 18 years: blood pressure ≥140/90 mmHg)
④New-onset thrombocytopenia (platelet count <50 × 109/L or a decrease in platelet count ≥50% from baseline)
⑤New-onset anemia (hemoglobin value below the lower limit of normal reference values or increased blood transfusion requirements)
⑥Evidence of microangiopathy (presence of fragmented red blood cells in peripheral blood or pathological examination results of tissue specimens indicating microangiopathy)
⑦Terminal complement activation (plasma sC5b-9 value above the upper limit of normal values for healthy individuals)

sC5b-9: Soluble complement membrane attack complex;①,②,③: Close monitoring is required for consideration of TA-TMA diagnosis;②+⑦: Indicates poor prognosis, suggesting early intervention should be considered.

**Figure 2:**
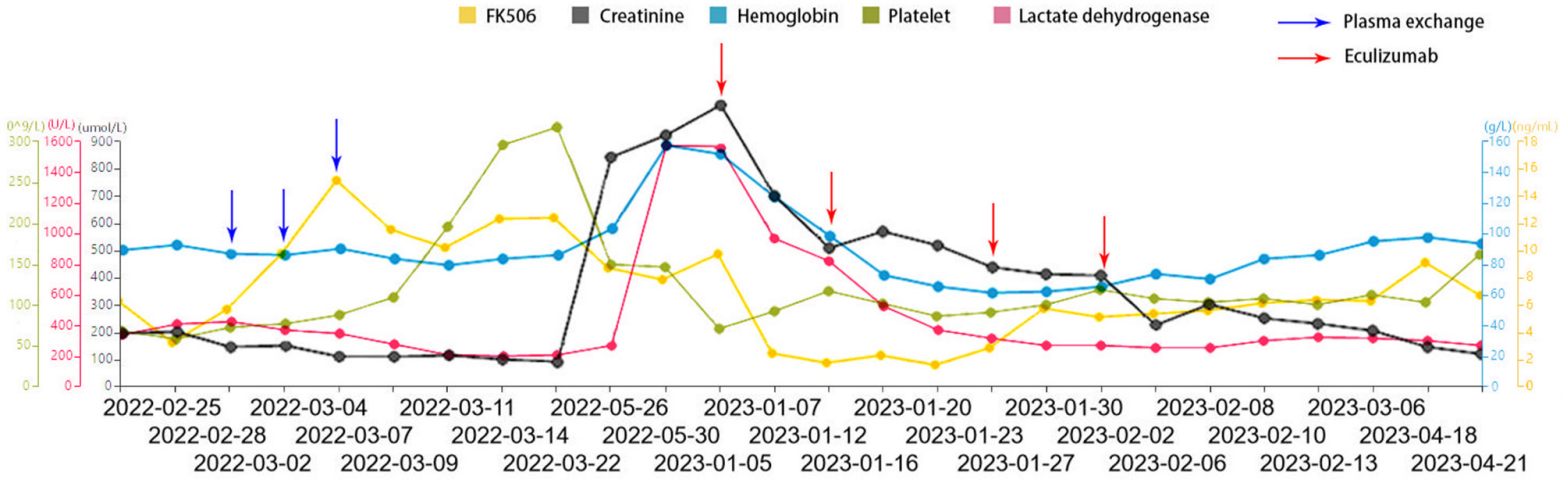
Changes in patient indices following transplantation. The patient, diagnosed with aHUS, initially underwent plasma exchange, which yielded limited efficacy. Subsequently, a treatment regimen with eculizumab was initiated, involving four doses of 900 mg each over a period of 3 weeks. This treatment led to a gradual recovery in urine output and significant improvements in hemoglobin and platelet counts, as well as reductions in creatinine and LDH levels.

The patient first received an induction regimen, with 900 mg of eculizumab administered weekly during the first 4 weeks of treatment (on January 7th, January 16th, January 27th, and February 3rd). Subsequently, in accordance with the instructions for using eculizumab, the patient underwent long-term maintenance therapy (receiving 900 mg of eculizumab every 2 weeks). However, due to personal reasons of the patient, the maintenance therapy was only continued for a period of 3 months. Nevertheless, during the follow-up after this period, it was found that the patient did not experience a recurrence of aHUS. After the therapy, there were significant clinical improvements, including normalization of blood pressure, hemoglobin, and LDH levels, as well as an increase in platelet count (Figure 2). Urine output improved to over 2,000 ml. Following discharge, the patient continued regular follow-up visits. Serum creatinine decreased to a minimum of 160 μmol/L, LDH to 232 U/L, and C5b-9 to 190 ng/ml. Eculizumab treatment resulted in decreased serum creatinine and LDH levels, increased urine output, and reduced inflammation, indicating a favorable response to the treatment regimen (Figure 2). The patient has been maintained under long-term follow-up, during which graft function has remained stable. Laboratory parameters, including platelet count, hemoglobin level, and lactate dehydrogenase concentration, have consistently remained within normal ranges (Supplementary Table 2). Furthermore, throughout a follow-up period exceeding 12 months since the initiation of therapy, no recurrence of aHUS-related manifestations has been observed.

## 3 Discussion

aHUS, a disorder stemming from dysregulation of the complement regulatory pathway, significantly impacts renal transplant recipients' prognosis through disease recurrence. This study presents a case of recurrent aHUS following kidney transplantation triggered by SARS-CoV-2 infection, with genetic analysis revealing pathogenic variants in the C3 and CFHR5 genes. The current report provides critical insights into the diagnosis and management of recurrent aHUS, emphasizing that therapeutic strategies should incorporate comprehensive genetic risk assessment and clinical progression monitoring. Individualized treatment approaches based on identified genetic predispositions and triggering factors are essential for optimizing long-term patient outcomes.

The *de novo* or recurrent aHUS in renal transplant recipients results from the combined effects of triggering factors and genetic or acquired dysregulation of the complement system. Major triggering factors include ischemia-reperfusion injury (IRI), antibody-mediated rejection (ABMR), and viral infections (5). These factors can lead to uncontrolled activation of the alternative complement pathway, resulting in endothelial cell injury, platelet aggregation, coagulation cascade activation, formation of microthrombi in renal arterioles, and erythrocyte destruction (11). The recurrence mechanism of aHUS is closely related to its timing. Early recurrence is often associated with perioperative IRI and endothelial toxicity induced by immunosuppressive agents, particularly calcineurin inhibitors such as tacrolimus (12). In this case, the patient experienced the first recurrence of aHUS on postoperative day 5, likely due to IRI during transplantation and high-dose tacrolimus administration in the early postoperative period, supporting this mechanism. In contrast, late recurrence is typically triggered by infections, graft dysfunction, or metabolic disorders, with persistent activation of the alternative complement pathway as the core mechanism (12). In this case, recurrence was induced by SARS-CoV-2 infection, likely involving cytokine storm-mediated inflammation and excessive activation of the alternative complement pathway, ultimately leading to aHUS relapse in a genetically susceptible individual. In recent years, an increasing number of case reports have described SARS-CoV-2 infection associated TMA in the absence of any other known etiologic factor or SARS-CoV-2 triggered aHUS recurrence. Notably, several of these reports have involved kidney transplant recipients. Collectively, these observations reinforce the association between SARS-CoV-2 infection and the development of TMA or aHUS (13–15). The present case report provides additional support for this link. The clinical features—including a prior history of aHUS, the presence of relevant genetic variants, and renal biopsy findings indicative of disease recurrence—along with the exclusion of other potential etiologies such as rejection or TTP, strongly suggest that the episode represented a reactivation of aHUS rather than a secondary form of TMA. This conclusion aligns with previous studies suggesting that viral infections can trigger endothelial injury and microvascular thrombosis through alternative complement pathway activation (16). Furthermore, while SARS-CoV-2 infection was the identified trigger here, it is important to acknowledge that other potent immune stimuli, including COVID-19 vaccination, have also been rarely reported as potential triggers for *de novo* TMA or aHUS relapse in susceptible individuals, including transplant recipients (17). Although vaccination status was not a factor in this specific recurrence, awareness of this potential trigger is essential for comprehensive patient management and risk assessment.

According to the standard treatment strategy for kidney transplantation in patients with aHUS, complement activation testing and relevant genetic analysis should be performed in patients with end-stage renal disease caused by TMA to assess the risk of recurrence. Variants in complement-related genes serve as the core basis for stratifying aHUS recurrence risk (18). Studies have shown that the recurrence rates in patients carrying mutations in CFH, CFI, C3, and MCP are 75%−80%, 70%−80%, 40%−50%, and 7.6%−20%, respectively. Meanwhile, mutations in CFB are associated with a 100% recurrence rate, though their incidence is relatively low (1%−4%) (19, 20). Among these, C3 mutations lead to sustained activation of the alternative complement pathway and are considered moderate-risk factors, whereas deletions or mutations in CFHR genes impair the regulatory function of complement factor H and are classified as high-risk markers (21). In this case, the patient carried mutations in both C3 and CFHR5, further underscoring the importance of pre-transplant genetic screening. Despite the patient's well-documented medical history, genetic testing was not conducted preoperatively, preventing timely implementation of prophylactic treatment. During the initial recurrence, in the absence of complement-targeted therapies, PE was administered, leading to clinical improvement, and the patient was closely monitored. However, a severe recurrence of aHUS, significantly compromising graft function, occurred following SARS-CoV-2 infection, a phenomenon also reported in the literature. Studies have demonstrated that prophylactic eculizumab administration before transplantation can effectively reduce post-transplant recurrence and significantly improve graft survival in high-risk patients (22). However, at the time of this patient's kidney transplant, our center lacked the necessary genetic testing and prophylactic treatment protocols. Consequently, the absence of a comprehensive preoperative evaluation and intervention resulted in post-transplant recurrence, leading to severe renal impairment. Although the patient showed improvement after receiving eculizumab upon recurrence, this case further validates the critical role of preoperative prophylaxis. Based on this experience, our center has since implemented genetic testing for subsequent patients, along with risk stratification and prophylactic interventions in accordance with international guidelines, and to date, no further aHUS recurrences have been observed.

Furthermore, continuous monitoring for aHUS recurrence after discontinuation of eculizumab therapy is crucial. Although CH50 and AH50 assays can be used to assess complement activity, their clinical utility is limited (23). A more reliable approach is to evaluate the deposition of C5b-9 on non-activated endothelial cells in serum, which provides a more accurate assessment of complement blockade and informs individualized dosing regimens (24). In cases of recurrence, prompt reinitiation of eculizumab therapy is warranted, while lifelong treatment may be necessary for patients at high risk of relapse. In this case, the patient developed end-stage renal failure due to genetic aHUS and experienced two post-transplant recurrences, highlighting the indispensability of lifelong complement blockade. Given that mutations in complement-related genes lead to persistent activation of the alternative complement pathway, conventional immunosuppressive therapy alone is insufficient to fully correct this dysregulation. Even in the presence of stable graft function, minor triggers such as infections can induce excessive activation of the terminal complement pathway, resulting in aHUS relapse (25). Therefore, temporary drug discontinuation or dose reduction may increase the risk of recurrence, necessitating long-term, and potentially lifelong, maintenance of eculizumab therapy. Despite challenges related to treatment cost and accessibility, the long-term benefits of sustained therapy outweigh the potential risks for high-risk patients.

## 4 Limitations

This case report has several limitations inherent to its design. Firstly, as a single case report, the findings cannot be generalized to the broader population of kidney transplant recipients or even all aHUS patients. The response observed here may not be representative. Secondly, while we have presented follow-up data extending beyond the initial 3 months post-recurrence treatment (now exceeding 12 months on maintenance eculizumab), the overall duration of observation remains relatively short for definitively assessing the very long-term graft survival and the absolute necessity of lifelong therapy, although current evidence strongly supports this treatment in high-risk genetic profiles. Thirdly, this case report primarily focuses on the effective treatment of aHUS recurrence with eculizumab. However, preoperative genetic screening and prophylactic treatment regimens for patients at high risk of aHUS recurrence are also necessary.

## 5 Conclusion

Deletions or mutations in complement-related genes, such as CFHR, are high-risk factors for aHUS recurrence, and early screening is of significant clinical value for risk assessment and intervention. Additionally, infections, exemplified by SARS-CoV-2, can trigger aHUS recurrence through complement activation, while eculizumab has been demonstrated to effectively control both *de novo* and recurrent aHUS. By integrating genetic risk stratification with targeted complement blockade strategies, aHUS may be transformed from a severe, transplant-threatening complication into a chronic, manageable condition, ultimately improving the prognosis of high-risk patients.

## Data Availability

All relevant data is contained within the article: The original contributions presented in the study are included in the article/supplementary material, further inquiries can be directed to the corresponding authors.

## References

[1] Chan L Chaudhary K Saha A Chauhan K Vaid A Zhao S. AKI in hospitalized patients with COVID-19. J Am Soc Nephrol. (2021) 32:151–60. 10.1681/ASN.2020050615 32883700 PMC7894657

[2] Noris M Benigni A Remuzzi G. The case of complement activation in COVID-19 multiorgan impact. Kidney Int. (2020) 98:314–22. 10.1016/j.kint.2020.05.013 32461141 PMC7246017

[3] Korotchaeva J Chebotareva N Andreeva E Sorokin Y McDonnell V Stolyarevich E. Thrombotic microangiopathy triggered by COVID-19: case reports. Nephron. (2022) 146:197–202. 10.1159/000520144 34808629 PMC8678244

[4] Epperla N Li A Logan B Fretham C Chhabra S Aljurf M. Incidence, risk factors for and outcomes of transplant-associated thrombotic microangiopathy. Br J Haematol. (2020) 189:1171–81. 10.1111/bjh.16457 32124435 PMC7726817

[5] Ávila A Gavela E Sancho A. Thrombotic microangiopathy after kidney transplantation: an underdiagnosed and potentially reversible entity. Front Med. (2021) 8:642864. 10.3389/fmed.2021.642864 33898482 PMC8063690

[6] Garg N Rennke HG Pavlakis M Zandi-Nejad K. De novo thrombotic microangiopathy after kidney transplantation. Transplant Rev (Orlando). (2018) 32:58–68. 10.1016/j.trre.2017.10.001 29157988

[7] Avila A Gavela E Sancho A. Thrombotic microangiopathy after kidney transplantation: an underdiagnosed and potentially reversible entity. Front Med. (2021) 8:642864. 10.3389/fmed.2021.642864 33898482 PMC8063690

[8] Azoulay E Knoebl P Garnacho-Montero J Rusinova K Galstian G Eggimann P. Expert statements on the standard of care in critically ill adult patients with atypical hemolytic uremic syndrome. Chest. (2017) 152:424–34. 10.1016/j.chest.2017.03.055 28442312

[9] Mannes M Dopler A Zolk O Lang SJ Halbgebauer R Höchsmann B. Complement inhibition at the level of C3 or C5: mechanistic reasons for ongoing terminal pathway activity. Blood. (2021) 137:443–55. 10.1182/blood.2020005959 33507296

[10] Tang ZC Hui H Shi C Chen X. New findings in preventing recurrence and improving renal function in AHUS patients after renal transplantation treated with eculizumab: a systemic review and meta-analyses. Ren Fail. (2023) 45:2231264. 10.1080/0886022X.2023.2231264 37563792 PMC10424606

[11] Imanifard Z Liguori L Remuzzi G TMA. in Kidney Transplantation. Transplantation. (2023) 107:2329–40. 10.1097/TP.0000000000004585 36944606

[12] Zuber J Fakhouri F Roumenina LT Loirat C Frémeaux-Bacchi V French Study Group for aHUS/C3G. Use of eculizumab for atypical haemolytic uraemic syndrome and C3 glomerulopathies. Nat Rev Nephrol. (2012) 8:643–57. 10.1038/nrneph.2012.214 23026949

[13] Akilesh S Nast CC Yamashita M Henriksen K Charu V Troxell ML. Multicenter clinicopathologic correlation of kidney biopsies performed in COVID-19 patients presenting with acute kidney injury or proteinuria. Am J Kidney Dis. (2021) 77:82–93.e1. 10.1053/j.ajkd.2020.10.001 33045255 PMC7546949

[14] Fahim P Nicolaysen A Yabu JM Zuckerman JE. Osmotic tubulopathy and acute thrombotic microangiopathy in a kidney transplant recipient with a breakthrough SARS-CoV-2 infection. Kidney Med. (2022) 4:100492. 10.1016/j.xkme.2022.100492 35637695 PMC9134749

[15] Tiwari V Bhandari G Gupta A Gupta P Bhargava V Malik M. Atypical HUS triggered by COVID-19: a case report. Indian J Nephrol. (2022) 32:367–70. 10.4103/ijn.ijn_196_21 35967527 PMC9364994

[16] Ahmadian E Hosseiniyan Khatibi SM Razi Soofiyani S Abediazar S Shoja MM Ardalan M. COVID-19 and kidney injury: Pathophysiology and molecular mechanisms. Rev Med Virol. (2021) 31:e2176. 10.1002/rmv.2176 33022818 PMC7646060

[17] Pattonieri EF Gregorini M Grignano MA Islami T D'Ambrosio G Ardissino G. Atypical hemolytic uremic syndrome associated with BNT162b2 mRNA COVID-19 vaccine in a kidney transplant recipient: a case report and literature review. Infect Dis Rep. (2025) 17:14. 10.3390/idr17010014 39997466 PMC11855336

[18] Vaisbich MH Andrade LG Barbosa MI Castro MC Miranda SM Poli-de-Figueiredo CE. Recommendations for diagnosis and treatment of Atypical Hemolytic Uremic Syndrome (aHUS): an expert consensus statement from the Rare Diseases Committee of the Brazilian Society of Nephrology (COMDORA-SBN). J Bras Nefrol. (2025) 47:e20240087. 39918340 10.1590/2175-8239-JBN-2024-0087en PMC11804885

[19] Feitz WJC van de Kar NCAJ Orth-Höller D van den Heuvel LPJW Licht C. The genetics of atypical hemolytic uremic syndrome. Med Genet. (2018) 30:400–9. 10.1007/s11825-018-0216-0 30930551 PMC6404389

[20] Abbas F El Kossi M Kim JJ Sharma A Halawa A. Thrombotic microangiopathy after renal transplantation: Current insights in de novo and recurrent disease. World J Transplant. (2018) 8:122–41. 10.5500/wjt.v8.i5.122 30211021 PMC6134269

[21] Zuber J Le Quintrec M Morris H Frémeaux-Bacchi V Loirat C Legendre C. Targeted strategies in the prevention and management of atypical HUS recurrence after kidney transplantation. Transplant Rev. (2013) 27:117–25. 10.1016/j.trre.2013.07.003 23937869

[22] Gonzalez Suarez ML Thongprayoon C Mao MA Leeaphorn N Bathini T Cheungpasitporn W. Outcomes of kidney transplant patients with atypical hemolytic uremic syndrome treated with eculizumab: a systematic review and meta-analysis. J Clin Med. (2019) 8:919. 10.3390/jcm8070919 31252541 PMC6679118

[23] Raina R Grewal MK Radhakrishnan Y Tatineni V DeCoy M Burke LL. Optimal management of atypical hemolytic uremic disease: challenges and solutions. Int J Nephrol Renovasc Dis. (2019) 12:183–204. 10.2147/IJNRD.S215370 31564951 PMC6732511

[24] Galbusera M Noris M Gastoldi S Bresin E Mele C Breno M. An ex vivo test of complement activation on endothelium for individualized eculizumab therapy in hemolytic uremic syndrome. Am J Kidney Dis. (2019) 74:56–72. 10.1053/j.ajkd.2018.11.012 30851964

[25] Benamu E Montoya JG. Infections associated with the use of eculizumab: recommendations for prevention and prophylaxis. Curr Opin Infect Dis. (2016) 29:319–29. 10.1097/QCO.0000000000000279 27257797

