# Supplementary material for: Case Report: Effective use of eculizumab in treating recurrent atypical HUS following renal transplantation triggered by SARS-CoV-2 infection
Source: Front Med (Lausanne). 2025 Nov 5;12:1515988. doi: 10.3389/fmed.2025.1515988 (PMC12627061; doi:10.3389/fmed.2025.1515988)
Supplement: Supplementary file 1 [file Data_Sheet_1.docx]

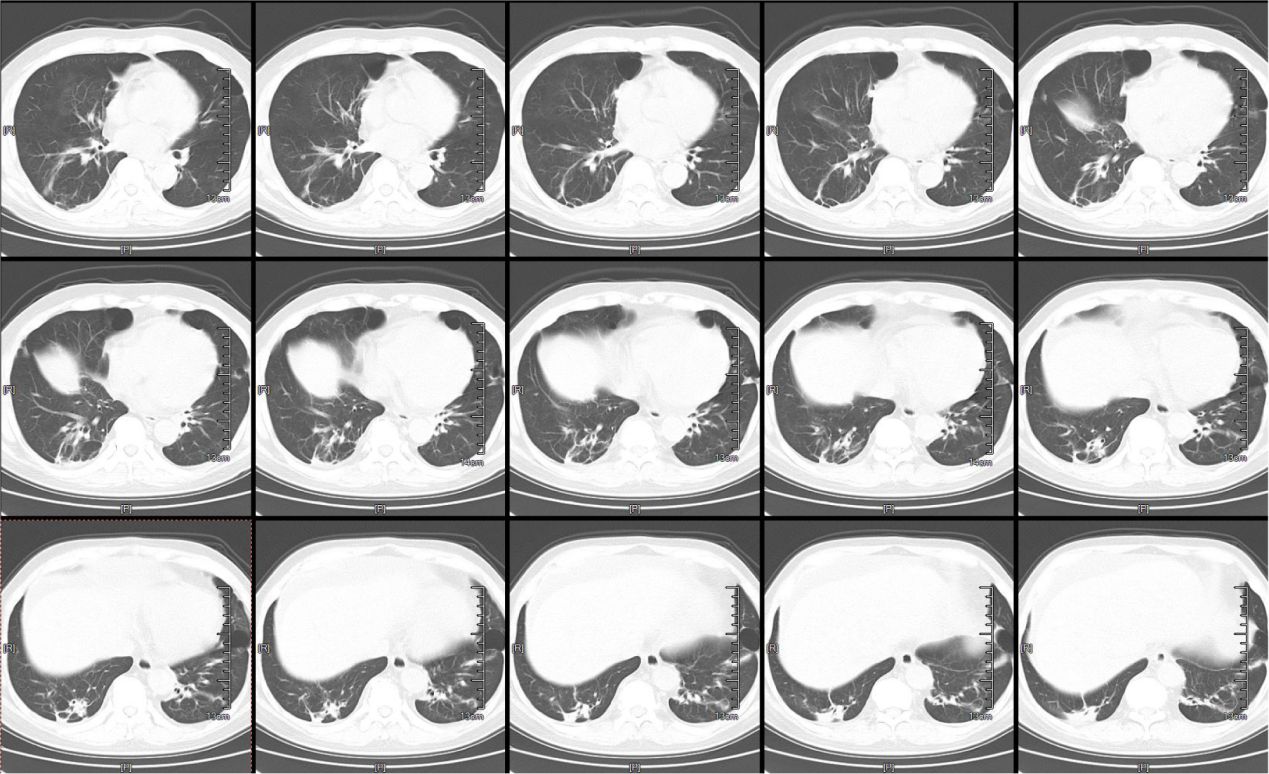


Supplementary Figure 1. The chest CT scan results of the patient reveal viral multi-segmental pneumonia.

The scan shows increased bilateral lung markings with patchy, slightly high-density shadows in the upper lobes of both lungs. Additionally, patchy and linear high-density shadows are observed in the lower lobes of both lungs. Irregular-shaped areas without lung markings are scattered throughout both lungs. The openings of the lung segments are unobstructed. No enlarged lymph nodes are present in the mediastinum or hilar regions. There are no abnormalities detected in the bilateral pleural cavities, although mild thickening of the bilateral pleura is noted.

| **Gene** | **Chromosome Location** | **Transcript Location** | **Nucleotide/**  **Amino Acid** | **Zygosity** | **Frequency in Normal Individuals** | **RS Number** | **Pathogenicity Analysis** | **Disease/Inheritance Pattern** |
| --- | --- | --- | --- | --- | --- | --- | --- | --- |
| C3 | chr19  6692971 | NM_00006  4.4 exon26 | c.3343G>A  P.D1115N^18^ | het | - | rs1219  09585 | Uncertain | 1.Complement C3 deficiency  (OMIM:613779). AR  2.Susceptibility to hemolytic uremic syndrome, type 5  (OMIM:612925). AD  3.Age-related macular degeneration 9  (OMIM:611378). - |
| CFHR5 | chr1  196994182 | NM_03078  7.4 exon4 | c.533A>G p.N178S^19,20^ | het | 0.000410247 | rs2004 27185 | Uncertain | CFHR5-deficient Nephropathy  (OMIM:61480 9).AD |

Supplementary Table 1 The patient's gene variants associated with TMA

References:

18.Frémeaux-Bacchi V, Miller EC, Liszewski MK, et al. Mutations in complement C3 predispose to development of atypical hemolytic uremic syndrome. Blood. 2008;112(13):4948-4952. doi:10.1182/blood-2008-01-133702

19.Zhai YL, Meng SJ, Zhu L, et al. Rare Variants in the Complement Factor H-Related Protein 5 Gene Contribute to Genetic Susceptibility to IgA Nephropathy. *J Am Soc Nephrol*. 2016;27(9):2894-2905. doi:10.1681/ASN.2015010012

20.Liu JW, Wang P, Huang J, et al. *Zhonghua Er Ke Za Zhi*. 2019;57(9):674-679. doi:10.3760/cma.j.issn.0578-1310.2019.09.006
