# Supplementary material for: Case Report: Effective use of eculizumab in treating recurrent atypical HUS following renal transplantation triggered by SARS-CoV-2 infection
Source: Front Med (Lausanne). 2025 Nov 5;12:1515988. doi: 10.3389/fmed.2025.1515988 (PMC12627061; doi:10.3389/fmed.2025.1515988)
Supplement: Supplementary file 2 [file Data_Sheet_2.docx]

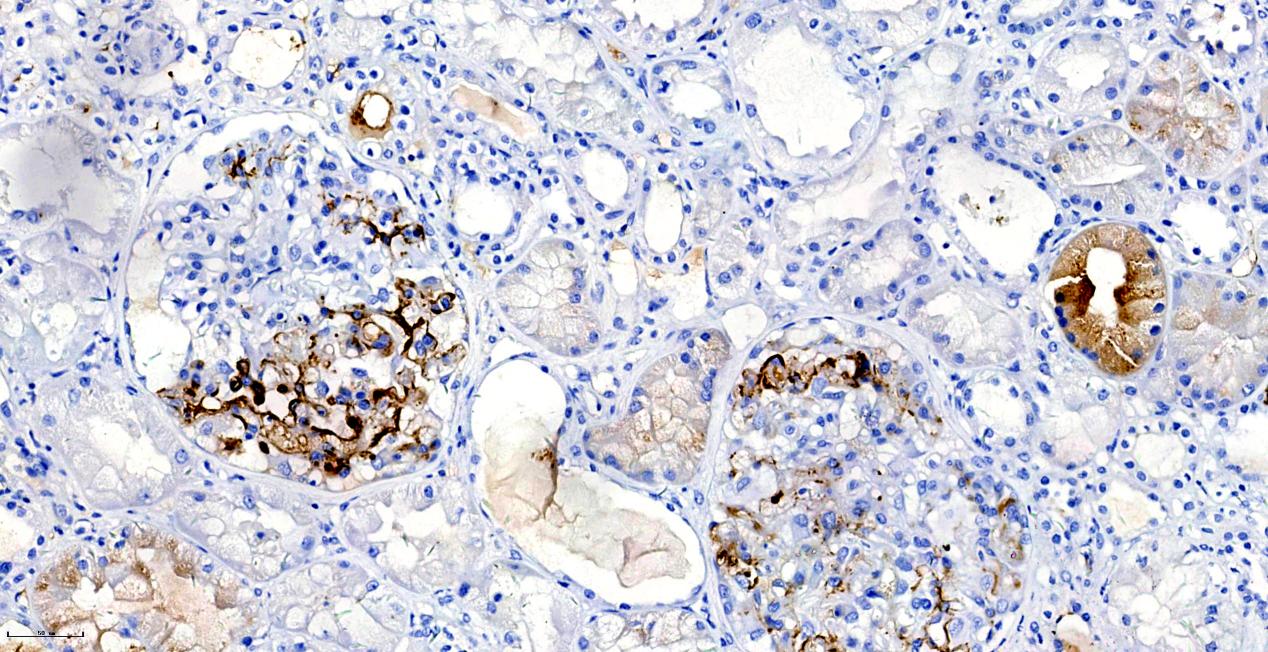


This patient's peritubular capillary C4d staining showed negative results, with the glomerular capillary loop serving as a positive control.
